# Familial Hemiplegic Migraine With Progressive Cerebellar Ataxia Caused by a p.Thr666Met CACNA1A Gene Mutation in a Chinese Family

**DOI:** 10.3389/fneur.2019.01221

**Published:** 2019-11-19

**Authors:** Mengmeng Li, Xiangyu Zheng, Rui Zhong, Qian Zhao, Yingxue Lu, Zan Wang, Weihong Lin

**Affiliations:** Department of Neurology, The First Hospital of Jilin University, Changchun, China

**Keywords:** familial hemiplegic migraine, migraine, genetic, CACNA1A gene, encephalopathy

## Abstract

Here, we describe the first case of familial hemiplegic migraine type 1 (FHM1) resulting from a T666M mutation in the CACNA1A gene of a Chinese individual. A 54-year-old female patient demonstrated extensive clinical manifestations, including transient paropsia, hemianesthesia, logaphasia, hemiplegia, migraine, fever, impaired consciousness, and progressive cerebellar ataxia. At admission, neurological examination showed a fever of 38.6°C, coma, bilateral pupillary constriction, left-sided deviation of both eyes, meningeal irritation, and bilateral positive Chaddock's sign. Brain magnetic resonance imaging (MRI) displayed only cerebellar atrophy. The pressure and white blood cells of the cerebrospinal fluid (CSF) were elevated. Her nine relatives also had similar clinical spectra. To further clarify the diagnosis, we conducted a genetic analysis on the family. The results of genetic testing showed that all seven living affected members carried the T666M mutation in the CACNA1A gene. This case report indicates that the diagnosis of FHM should be taken into account when a patient manifests migraine accompanied with hemiplegia, acute encephalopathy, and abnormal CSF. In addition, genetic testing is indispensable for the identification of some atypical attacks of hemiplegic migraine.

## Introduction

Familial hemiplegic migraine (FHM) is an infrequent autosomal-dominant subtype of migraine with motor aura (weakness) (1). Individuals with FHM are initially affected in the first or second decade of life (2). Transient visual, sensory, motor, aphasic, and basilar-type symptoms are frequent auras. Russel and Ducros (3) systematically reviewed published case reports of individuals diagnosed with hemiplegic migraine and summarized the clinical symptoms, which vary from attacks with single hemiplegic migraine to serious forms with repeated coma and sustained hemiparesis, progressive cerebellar ataxia, acute encephalopathy, epilepsy, fleeting blindness, or oligophrenia. Evidence from previous studies demonstrated that three genes have been associated with FHM, including CACNA1A (FHM1), ATP1A2 (FHM2), and SCN1A (FHM3) (4). Recent progress has indicated that the PRRT2 gene might be the fourth hemiplegic migraine gene (5). Each gene has multiple mutation types. To date, 18 missense mutations of the CACNA1A gene that lead to FHM1 have been discovered. T666M is the most common mutation (6, 7). Several patients with FHM1 with a T666M mutation have been reported worldwide. However, an FHM1 case caused by the T666M mutation has not yet been described in the Chinese population. Thus, we present the first FHM1 family with the T666M mutation of the CACNA1A gene in a Chinese population.

## Case Report

**Figure 1:**
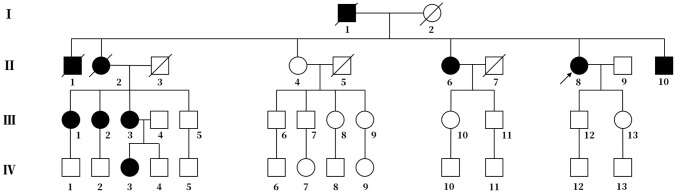
Pedigree chart of the family. The arrow represents the proband. Squares represent males, and circles represent females. The diagonal lines represent deceased family members. Black squares or circles indicate the members with FHM. White squares or circles represent members without FHM.

**Figure 2:**
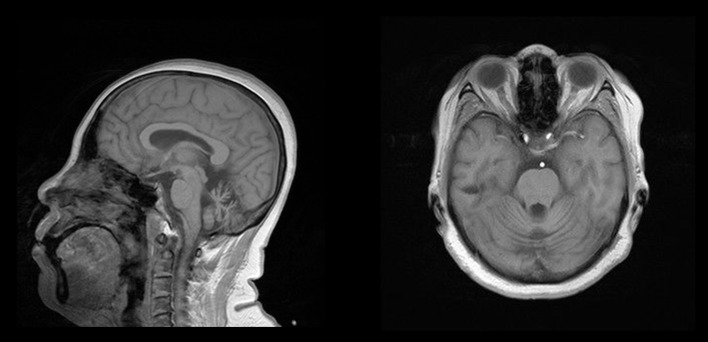
Brain MRI of the proband. Note the presence of obvious cerebellar atrophy.

The proband (Figure 1,II-8), a 54-year-old woman, could not walk unassisted until 48 months of age. She has had slow speech since childhood. The first migraine attack for this patient occurred at 40 years old, when she initially began seeing flickering light in her vision. Then, she experienced transient numbness in the left face, tongue, and limbs companied with aphasia. This patient then developed hemiplegia, which lasted for ~1 h. Subsequently, she experienced left throbbing headache, which vanished after enough rest for several hours. After the first episode, similar attacks occur 1 to 2 times per year. Sometimes she had agitation, hallucination, and somnolence for 2 to 3 days after the migraine according to her son. At age 45, her gait was ataxic and gradually worsened. Recently, a hemiplegic migraine occurred again, which was similar to a previous incident. However, she became agitated and hallucinatory along with a fever of 39.0°C the next day. Therefore, she was sent to a nearby hospital. Brain computed tomography (CT) showed cerebellar atrophy. Anti-fever medicine and antibiotic were administered. However, she had repeated fever and fell into a coma at that night. She was admitted to our hospital for continual coma on the third day. At admission, neurological examination indicated a fever of 38.6°C, coma, bilateral pupillary constriction, left-sided deviation of both eyes, meningeal irritation and bilateral positive Chaddock's sign. On the second day of admission, she became gradually conscious and achieved a normal body temperature. The diameter of the bilateral pupils and eye movement also recovered. However, her muscle strength was slightly weakened. Laboratory examination of cerebrospinal fluid (CSF) was abnormal (pressure 250 mmH_2_O, white cell count 18/μL). Brain MRI only exhibited cerebellar atrophy (Figure 2). Magnetic resonance angiography (MRA) of the brain, long-term video electroencephalogram (VEEG), and polysomnography showed no meaningful abnormalities. Because of acute symptoms and abnormal CSF, we suspected the diagnosis of viral encephalitis. Consequently, mannitol and acyclovir therapy were administered on the second day of admission. When she was discharged on the eleventh day, she demonstrated slow speech, unstable and wide-based gait, and mild limb ataxia. Luckily, other neurological examinations and CSF returned to normal.

The proband's deceased father and elder brother (Figure 1, I-1 and II-1) demonstrated slow speech, progressive unsteady gait, and typical hemiplegic migraine. They also experienced hallucination and sometimes coma. The attacks became increasingly severe with age. They suffered from left hemiparalysis for 1 year before their deaths.

The proband's eldest sister (Figure 1, II-2) also showed hemiplegic migraine, progressive ataxia and sluggish speech. She died of hemorrhage in the brain stem at age 53.

The proband's third elder sister (Figure 1, II-6), a 60-year-old female, had spoken slowly since her youth and started walking independently with insecure gait at age 13. The first typical hemiplegic migraine followed by drowsiness affected her at age 17. The cerebellar ataxia was more severe, and hemiplegic migraine became more frequent with age.

The proband's brother (Figure 1, II-10), a 50-year-old man, had slow speech and progressive ataxic gait since his childhood. He has experienced several hemiplegic migraines followed by drowsiness lasting for 2 to 3 days since the age of 20. The frequency of the episode increased with age.

The eldest daughter of the proband's eldest sister (Figure 1, III-1) is 51 years old. She possesses normal speech and gait. She began to show ephemeral hemidysesthesia, hemiplegia and dizziness at age 45.

The second daughter of the proband's eldest sister (Figure 1, III-2) is 49 years old. She has slow speech and normal gait. She had frequent attacks of hemiplegic migraine followed by vomiting when she was 8 years old. More attacks occurred with age.

Another daughter of the proband's eldest sister (Figure 1, III-3) is 47 years old. Her clinical performance is similar to that of her mother. She also suffered from more frequent attacks with age. Her daughter (Figure 1, IV-3), a 26-year-old girl, manifested temporary hemiplegic migraine and persistently slurred speech. She has needed a cane for walking assistance since childhood.

**Figure 3:**
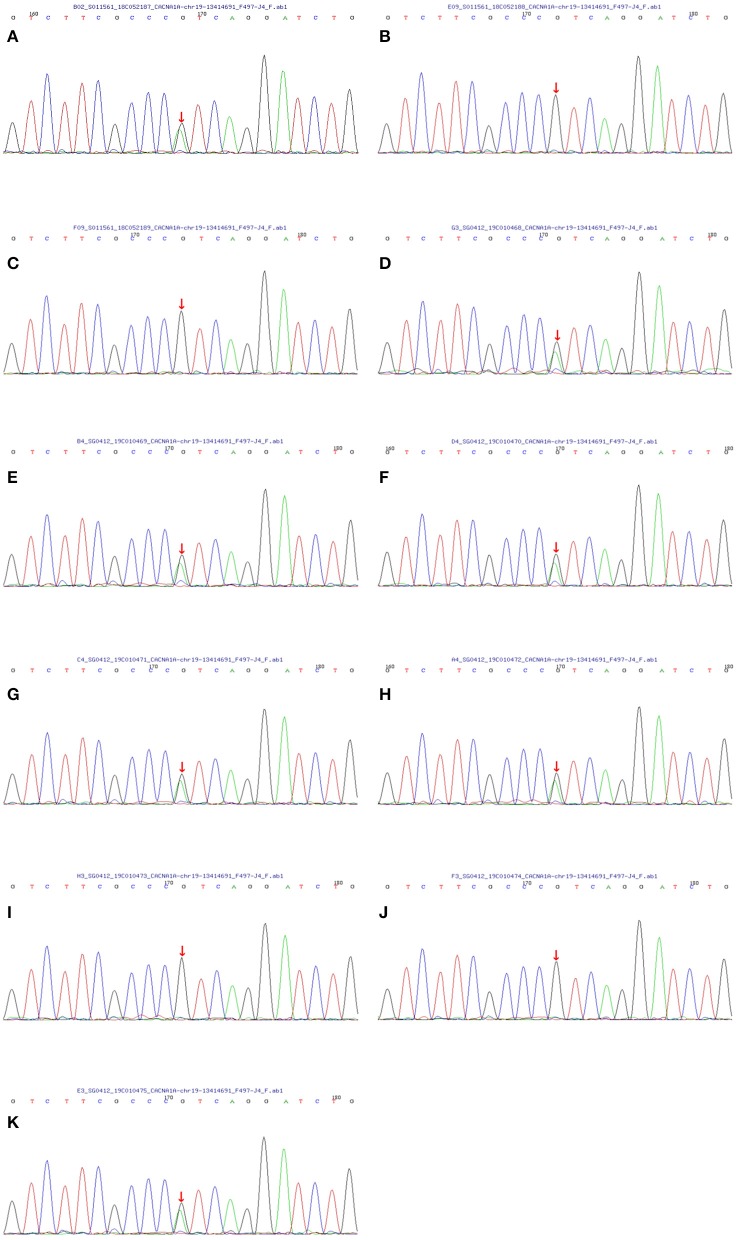
Sequencing map of the CACNA1A gene in the FHM family. All seven living members with FHM carried the T666M mutation in the CACNA1A gene (the C→ T replacement in exon 16 leads to athreonine-for-methionine substitution of amino acid 666). And four unaffected relatives did not contain the same mutation. The one-to-one correspondence between pictures and family members is that **(A)** matches the proband II-8, **(B)** matches III-12, **(C)** matches III-13, **(D)** matches II-10, **(E)** matches II-6, **(F)** matches III-1, **(G)** matches III-2, **(H)** matches III-3, **(I)** matches III-4, **(J)** matches IV-4 and **(K)** matches IV-3.

With the consent of seven living patients and four unaffected relatives in this family, we conducted a genetic analysis. The results revealed that all living patients with FHM carried the T666M mutation in CACNA1A genes. This pathogenic mutation was absent in four unaffected relatives (Figure 3).

## Discussion

Migraines are a prevalent and disabling headache disorder that affects 15 to 20% of the population. Hemiplegic migraine is a rare type of migraine with motor aura (weakness), and its prevalence is ~0.01%. When hemiplegic migraine affects at least one first or second-degree relative in a family, it is called FHM (2). FHM1, making up more than 50% of FHM cases, results from a mutation in the CACNA1A gene (8). The T666M mutation is the most common of all mutation types of the CACNA1A gene, occurring in 40% of FHM1 families (3). Here, we report a FHM1 family in China. There are 10 members with FHM in the family. They manifested mainly hemiplegic migraine, progressive cerebellar ataxia and sometimes acute encephalopathy. Genetic testing was performed on seven living patients with FHM and four unaffected relatives. The results showed that all seven living patients carried the T666M mutation, and the four unaffected relatives did not possess the T666M mutation in CACNA1A genes.

Although FHM1 cases caused by the T666M mutation in the CACNA1A gene have been reported in several countries, such as Japan and Korea (6, 9), this is the first Chinese FHM1 family to verify the T666M mutation of the CACNA1A gene. Ducros et al. (10, 11) proposed that the preponderance of T666M resulted from recurrent mutations instead of a founder effect, which was further supported by our case because it also existed in Chinese patients of a different ethnic background. Moreover, they also found that subjects with the T666M mutation had the highest penetrance of hemiplegic migraine (98 percent) and severe attacks with coma (50 percent) in FHM patients with cerebellar signs. These clinical characteristics were in line with the features of the affected patients in the family we described. All seven living patients carrying the T666M mutation complained of hemiplegic migraine (100 percent), three subjects demonstrated somnolence or coma (43 percent), and five affected members reported progressive cerebellar ataxia (71 percent). Typical attacks of hemiplegic migraine often begin in the first or second decade of life. Additionally, the frequency of attacks decreases with age according to a previous study (2). However, there were two patients whose initial attacks of hemiplegic migraine occurred in their fourth decade, and six patients experienced more frequent attacks with age in this family, which was different from the previous study. These differences are probably due to genetic expression, which is affected by other genes, the environment, hormones, etc.

FHM1 has been identified as an ion channel disease caused by CACNA1A gene mutations. The CACNA1A gene encodes the pore-forming α1A subunit of P/Q-type voltage-gate CaV2.1 calcium channels that are dominant calcium channels in Purkinje cells (P-type currents) and are highly present in cerebellar granule cells (P- and Q-type currents), which may be associated with a high incidence of cerebellar atrophy in FHM1 patients. P/Q-type voltage-gate CaV2.1 calcium channels are situated in the presynaptic terminal of glutamatergic and GABAergic neurons. Once an action potential arrives at the presynaptic terminal, CaV2.1 channels open rapidly, which permits Ca^2+^ to enter, then causes vesicles to fuse and glutamate to be released, leading to the following activation of postsynaptic receptors and generation of action potential. According to most cellular studies, FHM1 mutations can change the voltage-dependence of neuronal CaV2.1 channels toward more negative membrane potentials and increase the open probability of a channel, causing gain-of-function effects. FHM1 mutations exert the gain-of-function effect only on CaV2.1 glutamatergic channels rather than GABAergic terminals, which enhances neuronal excitability and decreases the threshold of cortical spreading depression, resulting in the occurrence of hemiplegic migraine (10, 12).

FHM accompanied by acute encephalopathy is rare and easily misdiagnosed. Patients often experience lumbar puncture to assess encephalomenigitis or subarachnoid hemorrhage due to meningeal irritation and pyrexia (13). Although several cases of hemiplegic migraine accompanied with encephalopathy have been described, the change in CSF composition is conflicting among previous studies. Tashiro et al. (14) reported an FHM1 family with recurrent encephalopathy and hemicerebral atrophy, and no abnormalities were observed in the CSF of the proband. However, Fitzsimons and Wolfenden (15) described an FHM family and found that the CSF leucocyte counts of their patients changed over time. Thus, they proposed that the different reports about the CSF composition of hemiplegic migraine may be due to the timing of the lumbar puncture to a certain extent. The CSF of the proband we reported showed elevated leucocyte counts on the second day, which returned to normal on the eleventh day of admission, which was consistent with Fitzsimons et al. The mechanism of hemiplegic migraine accompanied by acute encephalopathy is unknown. Fever may be central. In our case report, the proband had a sudden onset of fever without a clear history of infection, and neither antibiotic nor antipyretic was effective for her. One hypothesis indicated that fever and meningeal irritation are attributed to disorder of the microcirculation in the meningeal venous sinuses (13, 16). A recent investigation found that serum interleukin-6 and other inflammatory mediators were increased in patients with migraine. Elevated interleukin-6 in CSF was also reported. These findings support another hypothesis that inflammatory mediators may be involved in encephalopathy by destroying the blood-brain barrier in FHM (17, 18). In addition, according to some previous reports, MRI of individuals with FHM during attacks have shown hemispheric or lobar gyral swelling and restricted diffusivity. These changes of MRI indicate the occurrence of hemispheric cytotoxic edema during severe attacks of FHM, which may also be related to cerebral dysfunction and encephalopathy (18–21). Therefore, we highlight the request to carry out more studies to investigate the potential mechanism of acute encephalopathy in FHM. Hemiplegic migraine-related encephalopathy often seems to be self-limiting. However, it is fatal in a few cases (22). Therefore, we advise patients with FHM to be hospitalized when they present with acute encephalopathy, including a fever, psychiatric symptoms, coma, etc.

In conclusion, this is the first family with FHM1 caused by the T666M mutation of the CACNA1A gene in China. It is generally known that the clinical manifestations resulting from the same gene mutation are distinct under different genetic backgrounds and environmental factors. Our case report is aimed at enriching ethnic backgrounds about FHM1 mutations in the available databases and facilitating studies about the potential pathophysiological mechanisms of FHM1. The clinical features of FHM are complex and varied. When a patient experiences migraines accompanied with hemiplegia, acute encephalopathy and abnormal CSF, the diagnosis of FHM should be considered. In addition, genetic testing should be carried out in some cases, for example, when evident delay of walking or progressive cerebellar ataxia occurs as initial symptoms. Genetic analysis is helpful for doctors to judge the atypical manifestations of hemiplegic migraine and give proper treatments to patients in a timely manner, which may delay the development of hemiplegic migraine and improve the quality of life for patients.

## Ethics Statement

We obtained written informed consent of all subjects prior to genetic testing and for publication of this case report. There is no experimental intervention in this case report. Therefore, formal ethics approval is not needed.

## Author Contributions

WL put forward the idea and revised the manuscript. ML and RZ collected all the data and wrote the manuscript. ZW and XZ reviewed all the literature and revised the manuscript. QZ and YL analyzed and interpreted brain imaging and gene sequencing results. All authors approved the submitted version.

### Conflict of Interest

The authors declare that the research was conducted in the absence of any commercial or financial relationships that could be construed as a potential conflict of interest.
